# Retrospective analysis of 331 acute appendicitis patients: how appendicolith and CT features aid in differentiating complicated vs. uncomplicated appendicitis

**DOI:** 10.1186/s12876-025-04537-z

**Published:** 2025-12-13

**Authors:** Dawei Zhang, Shengqiang Wang, Haoyang Li, Zixuan Fu, Jing Zhang, Zhen Liu, Shikuan Li

**Affiliations:** 1 Department of Gastrointestinal and Hernia Surgery, Affiliated Hospital of Shandong University of Traditional Chinese Medicine, Jinan, Shandong China; 2 Department of Emergency General Surgery, The Affiliated Hospital of Qingdao University, No.16 Jiangsu Road, Shinan District, Qingdao, Shandong China; 3 Department of Emergency General Surgery, Jining No.1 People’s Hospital, Jining, Shandong China; 4 Department of Gastrointestinal Surgery, The Affiliated Hospital of Qingdao University, Qingdao, Shandong China; 5 Department of Radiology, The Affiliated Hospital of Shandong Second Medical University, Weifang, Shandong China

**Keywords:** Acute appendicitis, Appendicolith, Computed tomography, Retrospective studies

## Abstract

**Purpose:**

Appendicoliths are one of the important causes of acute appendicitis. Currently, there is no consensus on the relationship between appendicoliths and complicated appendicitis, and opinions on the treatment of appendicolith-associated appendicitis vary. This study aims to determine the significance of appendicolith in acute complicated appendicitis and to assess the characteristics of appendicoliths and Computed tomography (CT) features associated with complicated appendicitis.

**Methods:**

A retrospective analysis was conducted on patients who underwent surgical treatment for acute appendicitis at the affiliated hospital of Qingdao University from January 2016 to October 2023. Acute appendicitis was classified into two groups with and without appendicolith based on CT findings, intraoperative observations, and postoperative pathology. The clinical data of the two groups were analyzed and compared. Further subgroup analysis was performed within the appendicolith group based on pathological findings, comparing the location, size, and number of appendicoliths, as well as the length, diameter, and CT features of the appendix.

**Results:**

Among 331 patients with acute appendicitis, 179 had appendicolith, of which 106 were complicated appendicitis and 73 were uncomplicated appendicitis. Among 152 patients without appendicolith, 44 had complicated appendicitis and 108 had uncomplicated appendicitis. appendicoliths were independently associated with complicated appendicitis (OR = 1.88, 95% CI: 1.04–3.40, *p* = 0.036). In patients with appendicolith appendicitis, three factors were independently associated with complicated appendicitis: appendiceal diameter (OR = 1.20; 95% CI: 1.03–1.40), moderate-severe fat stranding (OR = 17.61; 95% CI: 3.19–97.33), and periappendiceal air (OR = 9.78; 95% CI: 1.17–81.46).

**Conclusion:**

Appendiceal appendicoliths are closely related to acute complicated appendicitis. The diameter of the appendix and moderate-severe fat stranding, periappendiceal air on CT are significant indicators for identifying complicated appendicitis in acute appendicitis with appendicolith.

## Introduction

Acute appendicitis is one of the most common surgical emergencies, with an annual incidence rate of 96.5–100 per 100,000 individuals [1]. Luminal obstruction [1] is one of the most common causes of appendicitis, including appendicolith obstruction [2], lymphoid follicle hyperplasia [3], parasitic infection, and, less commonly, appendiceal or cecal tumors. Lymphoid follicles atrophy with age, making lymphoid follicle hyperplasia more common in children, whereas appendicolith obstruction is more common in adults.

An appendicolith is a calcified material within the appendix observed on imaging studies. Studies have shown that approximately 3%−4% of asymptomatic patients incidentally discover appendicolith on CT. Pasty stools are more likely to form appendicoliths, and higher stool water content leads to fewer appendicoliths. The fiber content in the diet is a primary factor determining stool water content and viscosity. Some authors suggest that the higher obesity rates in developed countries compared to developing countries, along with low-fiber diets, lead to appendicoliths formation. In individuals with low-fiber diets, appendicoliths develop faster. High-fiber diets accelerate stool transit time, reduce stool viscosity, and inhibit appendicolith formation [4]. Once an appendicolith forms, it may gradually enlarge, with substances entering the appendiceal lumen adhering to the appendicolith. The expansion of previously negligible appendicoliths can eventually obstruct the lumen outflow and lead to acute appendicitis [2]. Approximately 20%−40% of patients with acute appendicitis are found to have appendicoliths on CT [2, 3, 5].

Based on gross and microscopic findings, appendicitis can be categorized into acute intraluminal inflammation, acute mucosal/submucosal inflammation, suppurative/phlegmonous appendicitis, gangrenous appendicitis, perforated appendicitis, and periappendiceal abscess [1, 6]. Clinically, appendicitis is classified as uncomplicated or complicated based on whether the appendix is perforated or at risk of perforation. Uncomplicated appendicitis includes acute intraluminal inflammation, acute mucosal/submucosal inflammation, and suppurative appendicitis. Complicated appendicitis includes gangrenous appendicitis, perforated appendicitis, and periappendiceal abscess [1, 6]. Surgical removal is the traditional treatment for appendicitis, especially in cases of conservative failure or suspected gangrenous or perforated appendicitis. However, nonoperative management (NOM) with antibiotic therapy has recently emerged as an alternative treatment strategy for uncomplicated appendicitis (i.e., those without gangrene or perforation) [7]. International guidelines from the American Association for the Surgery of Trauma, the UK National Institute for Health and Care Excellence (NICE), the World Society of Emergency Surgery, and the American College of Surgeons endorse the feasibility of treating uncomplicated appendicitis solely with antibiotics [8–10]. However, there is still controversy regarding the treatment of uncomplicated appendicitis with appendicoliths.

Appendicitis with appendicolith is generally considered related to complicated appendicitis [3, 11–14]. Appendicoliths are considered factors for the failure of nonoperative management or recurrent appendicitis in uncomplicated cases [15–18]. However, for many individuals, appendicoliths are incidental findings, with varying proportions (3%−25%) discovered by CT and autopsy in patients without appendicitis [5, 19, 20]. The appendicolith is an incidental finding and not always the primary cause of acute (nonperforated) appendicitis or gangrenous (perforated) appendicitis [20]. Despite numerous studies on appendicolith, their variability is significant, and research on their characteristics remains limited. This study compares the clinical presentation, laboratory tests, and histopathological classification of appendicitis with and without appendicolith treated surgically. Further, it compares the features of complicated and uncomplicated appendicitis within the appendicolith group, analyzing the role of appendicoliths in the progression of acute appendicitis to guide clinical diagnosis and treatment.

## Materials and methods

### Study design and case selection

**Fig. 1:**
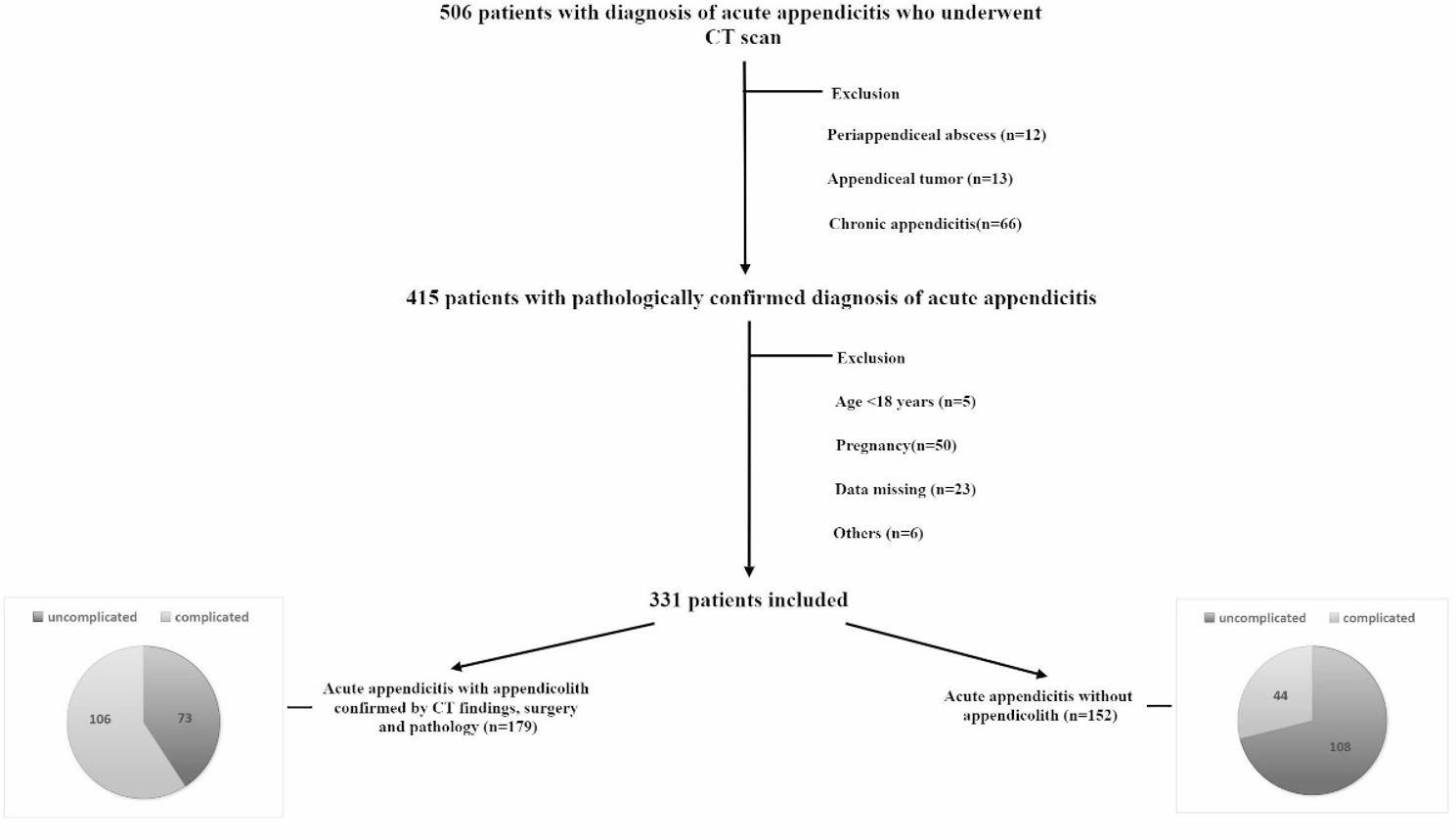
Flowchart of the patients

This study retrospectively analyzed the clinical and imaging data from 506 appendicitis patients that underwent appendectomy between January 2016 to December 2023 at the affiliated hospital of Qingdao University. The flowchart displaying the details regarding patient enrollment in this study is shown in Fig. 1. This study was approved by the dual Institution’s Ethics Committee, the requirement for informed consent was waived because of the retrospective nature of this study. Following were the patient inclusion criteria: (1) Age > 18 years; (2) Patients with appendicitis confirmed by CT and undergoing surgery; (3) Complete data (preoperative CT plain scan imaging, surgical records, pathology, etc.). Following were the patient exclusion criteria: (1) Appendix tumor; (2) Patients with periappendiceal abscess without operation; (3) Chronic appendicitis; (4) Appendicitis during pregnancy; (5) Incomplete clinic data.

### Clinical characteristics, image collection and definitions of CT findings

Demographic data, hospital length of stay, signs and symptoms, laboratory data, Alvarado score, and operative and pathological results were collected from the electronic medical records.

A 64-slice CT machine (IQon Spectral, Philips or Discovery CT750, GE Medical Systems or other CT scanners in the dual center) was utilized for scanning. All the patients were placed in the supine position. The scanning range was from the top of the diaphragm to the plane of the Symphysis pubis. The following conditions were used for CT scanning: (a) Tube current: 200 to 260 mA; (b) tube voltage: 120 kV; and (c) matrix: 512 × 512.

Two senior radiologists, with 18 and 21 years of experience respectively, independently reviewed CT scans of all patients and they reached a consensus through consultation when opinions differ. CT findings include appendiceal/periappendiceal, characteristics and appendicolith characteristics. The appendiceal/periappendiceal findings include appendix diameter, appendix length, fat stranding, periappendiceal air, periappendiceal fluid and small bowel dilatation. Appendicolith characteristics include the number, position and maximum diameter of the appendicolith. The definitions of each CT finding and measurements are provided in Table 1 [6, 21, 22].

**Table 1:** Definitions of CT findings and measurements

Findings	Definition
Appendiceal diameter	The maximum outer-to-outer diameter of the appendix, measured perpendicular to the longitudinal axis of the appendix on axial CT images.
Appendiceal length	The length of the appendix from its root to the blind end, measured on sagittal or coronal CT images.
Fat stranding	Increased attenuation of fat surrounding appendix. Just perceptible (thickness, 1–2 mm), mild; others, moderate–severe.
Extraluminal fluid	Fluid around the appendix without encapsulation. Minute periappendiceal(<5 mm); Little periappendiceal(5–20 mm); Large periappendiceal(>20 mm).
Periappendiceal air	Black bubble shadow outside the wall of appendiceal.
Small bowel dilatation	Small bowel caliber of larger than 2.5 cm.
Appendicolith	A hyperattenuating foci with a diameter > 2 mm located either inside the appendiceal lumen or outside in fluid or fluid collection.
Obstructive appendicolith	An appendicolith where the diameter of the appendix distal to the appendicolith dilates to be equal to or larger than the diameter of the appendicolith.
Appendicolith location	Classified by its position relative to the length of the appendix: proximal (proximal 1/3 of the appendix); middle (middle 1/3 of the appendix); distal (distal 1/3 of the appendix).
Appendicolith number	Single; multiple (for patients with > 1 appendicolith, the one with the largest maximum diameter is used as the representative of all appendicoliths in that patient); sludge-like (ill-defined flocculent or punctate slightly hyperattenuating foci within the appendiceal lumen).
Appendicolith length	The maximum diameter of the appendicolith, measured on axial CT images.

### Definitions

The presence of an appendicolith was identified by at least one of three methods: pre-operative CT scan, intraoperative identification, or histopathology report. Complicated appendicitis was defined as appendicitis with perforation, gangrene and/or periappendicular abscess formation.

### Statistical analysis

Descriptive statistics were used to analyze both qualitative and quantitative data. Categorical variables were presented as numbers or percentages, while continuous data were reported as either mean (standard deviation) or median (range) depending on their normal or skewed distribution.

Inferential statistics were employed to compare the differences between the two groups (patients without vs. with appendicolith, complicated vs. uncomplicated appendicitis). The Pearson chi-square test, or Fisher exact test was utilized for categorical variables, and the independent-sample t-test or Mann–Whitney U test was used for continuous variables with means or medians, respectively. Univariate and multivariate analyses were performed. Logistic regression was used to determine the odds ratio for independent predictors.

All analyses were performed using the IBM SPSS Statistics for Windows Version 26.0 and considering a statistical significance of a p value less than 0.05.

## Results

**Fig. 2:**
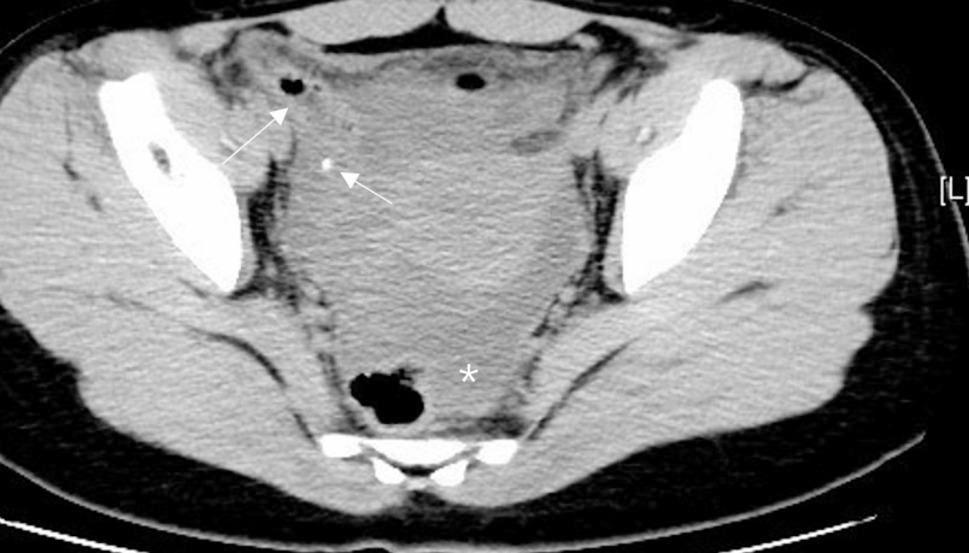
Extraluminal appendicolith and fluid. Axial CT images show an enlarged appendix with periappendiceal appendicolith (arrow) and large periappendiceal fluid (asterisk)

**Fig. 3:**
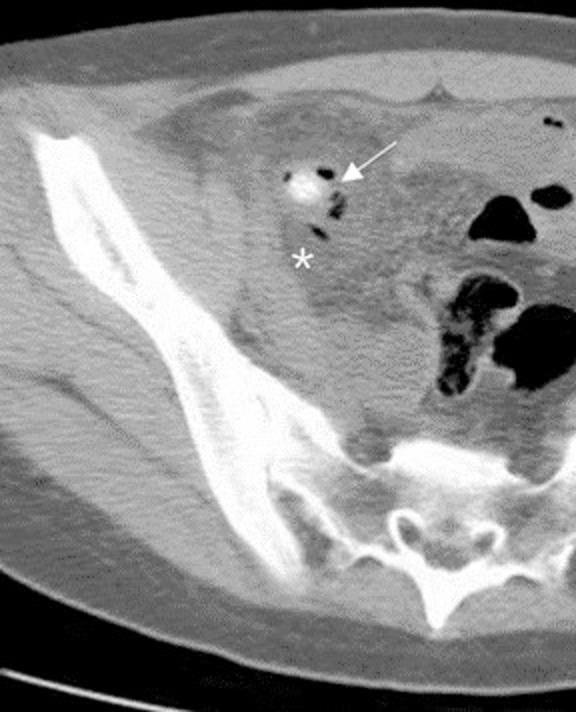
Periappendiceal air and extraluminal fluid. Axial CT images show an extraluminal air bubbles (arrow) mixed with fluid (asterisk) around the appendicolith

**Fig. 4:**
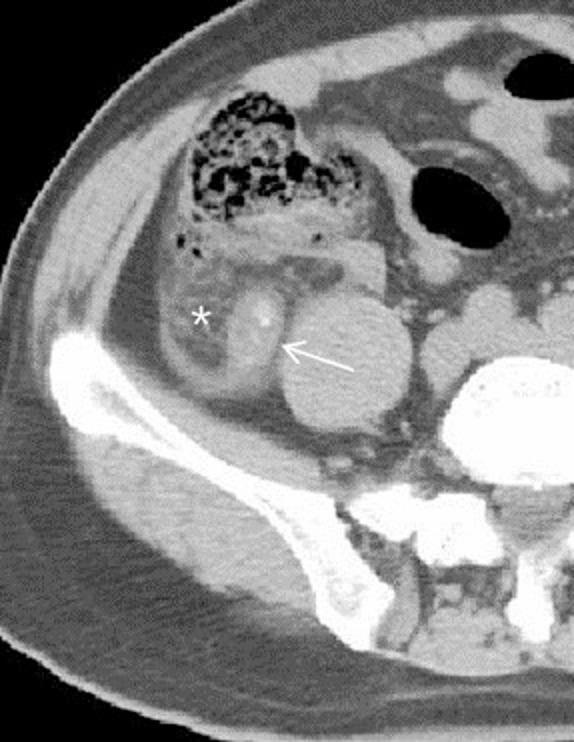
Moderate–severe fat stranding. Axial CT images show an enlarged appendix with appendicolith (arrow) and moderate–severe fat stranding (asterisk)

**Fig. 5:**
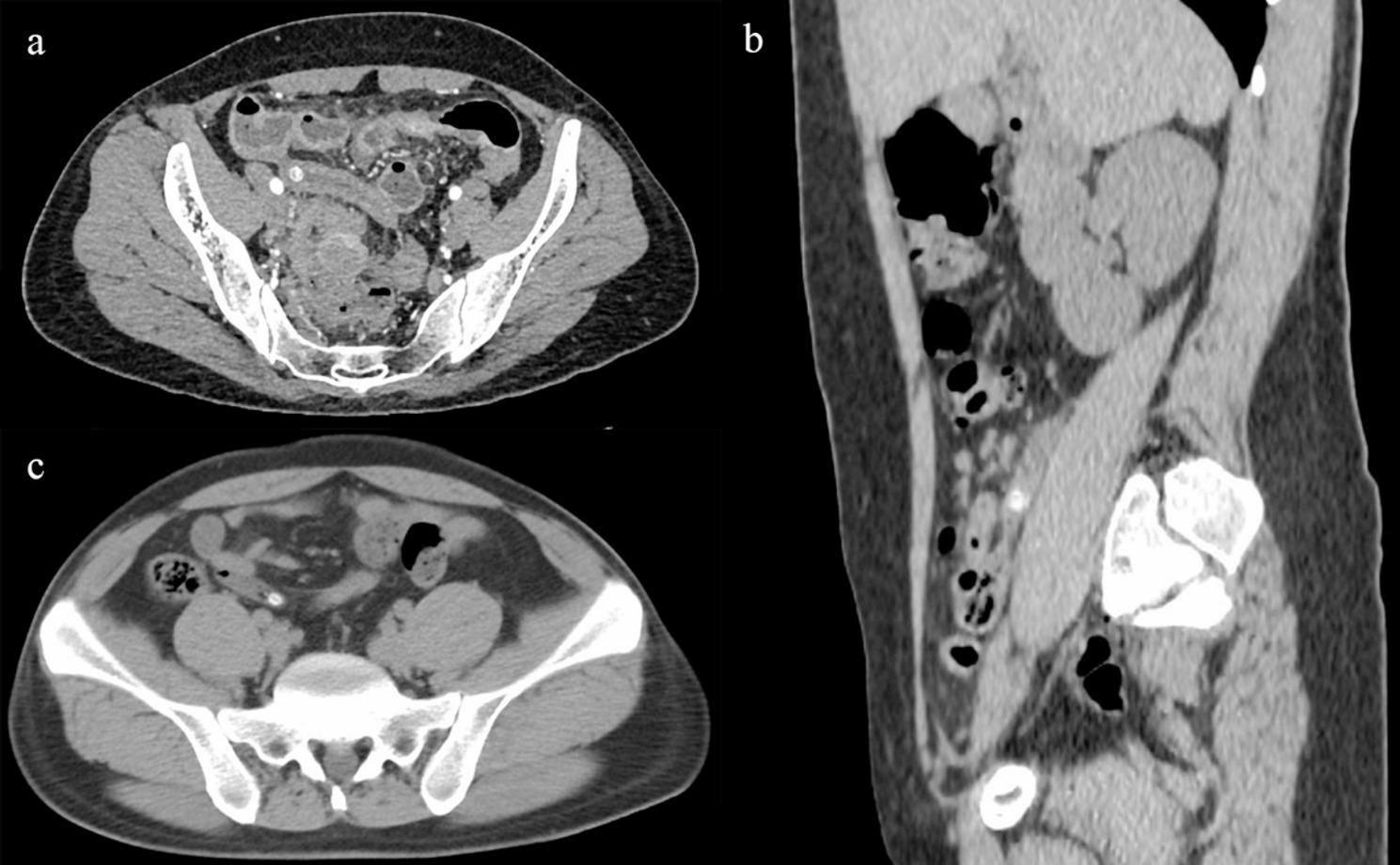
**a** Appendicolith in the proximal part of the appendix; **b** Appendicolith in the middle part of the appendix; **c** Appendicolith in the distal end of the appendix

A total of 331 patients were included in the study, divided into those with appendicoliths (*n* = 179) and those without (*n* = 152). Notably, 26 patients (14.5% of the appendicolith-positive group) had appendicoliths undetected by preoperative CT but confirmed intraoperatively/pathologically. The analysis focused on identifying factors associated with the presence of appendicoliths and complicated appendicitis. The details of CT findings are demonstrated in Table 1 and illustrated in Figs. 2, 3, 4 and 5.

There was no statistically significant difference in patient characteristics between the appendicitis with and without appendicolith in terms of age, gender, BMI, duration of symptom, hospital length of stay, postoperative hospital stays and surgical method (Table 2).

**Table 2:** Patient characteristics between those with and without appendicoliths

Variables	Total (*n* = 331)	Without appendicolith (*n* = 152)	With appendicolith (*n* = 179)	Statistic	*P*
Age (years), Mean ± SD	43.93 ± 18.59	45.69 ± 18.90	42.44 ± 18.23	t = 1.59	0.113
Gender, n (%)				χ²=0.25	0.617
Female	153 (46.22)	68 (44.74)	85 (47.49)		
Male	178 (53.78)	84 (55.26)	94 (52.51)		
BMI(Kg/m^2^), Mean ± SD	23.78 ± 3.54	24.05 ± 3.19	23.57 ± 3.80	t = 1.18	0.241
Symptoms and sings					
Migration of pain, n (%)	157 (47.72)	62 (41.06)	95 (53.37)	χ²=4.96	0.026
Anorexia, n (%)	278 (84.50)	122 (80.79)	156 (87.64)	χ²=2.92	0.087
Nausea or vomiting, n (%)	166 (50.46)	61 (40.40)	105 (58.99)	χ²=11.30	< 0.001
RLQ tenderness, n (%)	310 (94.22)	141 (93.38)	169 (94.94)	χ²=0.37	0.544
Rebound tenderness, n (%)	220 (66.87)	95 (62.91)	125 (70.22)	χ²=1.97	0.160
Temperature (°C), Mean ± SD	37.25 ± 1.00	37.23 ± 1.03	37.27 ± 0.98	t=−0.40	0.692
Duration of symptom (h), Mean ± SD	2.19 ± 1.88	2.18 ± 1.82	2.20 ± 1.93	t=−0.09	0.928
Labs (Mean ± SD)					
WBC count (cells/mm ^3^)	11,800 ± 4610	10,790 ± 4550	12,660 ± 4500	t=−3.75	< 0.001
Neutrophil count (cells/mm ^3^)	9720 ± 4540	8570 ± 4520	10,710 ± 4330	t=−4.39	< 0.001
Lymphocyte count (cells/mm ^3^)	1410 ± 1020	1520 ± 1050	1320 ± 990	t = 1.80	0.073
Neutrophilia (%)	80.34 ± 11.90	76.96 ± 13.68	83.21 ± 9.26	t=−4.78	< 0.001
Hemoglobin (g/L)	133.35 ± 15.99	134.25 ± 13.66	132.59 ± 17.73	t = 0.96	0.336
Platelets (10^9^/L)	222.77 ± 67.83	223.17 ± 73.49	222.44 ± 62.83	t = 0.10	0.923
Procalcitonin (ng/ml)	5.65 ± 11.09	6.24 ± 13.94	5.15 ± 7.93	t = 0.89	0.374
CRP (mg/L)	59.49 ± 68.88	51.23 ± 60.44	66.50 ± 74.76	t=−2.05	0.041
ALP (U/L)	63.96 ± 22.37	64.81 ± 20.62	63.23 ± 23.79	t = 0.64	0.525
AST (U/L)	22.94 ± 13.04	24.49 ± 15.96	21.62 ± 9.76	t = 2.00	0.046
ALT (U/L)	24.67 ± 19.84	26.33 ± 25.33	23.26 ± 13.46	t = 1.41	0.160
TBL (µmol/L)	64.25 ± 717.45	23.01 ± 15.85	99.28 ± 975.38	t=−0.96	0.336
Sodium (mmol/L)	139.61 ± 8.11	140.67 ± 2.85	138.71 ± 10.65	t = 2.20	0.028
Potassium (mmol/L)	4.50 ± 7.45	4.07 ± 0.40	4.87 ± 10.13	t=−0.97	0.335
Chlorine (mmol/L)	103.73 ± 8.51	104.11 ± 8.89	103.41 ± 8.18	t = 0.74	0.458
Albumin (g/L)	41.65 ± 7.47	42.51 ± 6.51	40.93 ± 8.14	t = 1.97	0.050
D-dimer (ng/ml)	771.29 ± 1228.86	816.39 ± 1592.78	732.99 ± 802.65	t = 0.61	0.539
CRP/Albumin	1.70 ± 2.26	1.42 ± 2.05	1.94 ± 2.41	t=−2.10	0.037
NLR	10.76 ± 9.51	9.31 ± 10.29	11.98 ± 8.62	t=−2.57	0.011
Alvarado score, Mean ± SD	6.69 ± 2.18	6.14 ± 2.13	7.16 ± 2.12	t=−4.31	< 0.001
HLOS (days), Mean ± SD	4.63 ± 4.01	4.51 ± 3.98	4.73 ± 4.05	t=−0.51	0.610
Postoperative hospital stays (days), Mean ± SD	4.23 ± 3.91	3.90 ± 3.73	4.51 ± 4.05	t=−1.41	0.159
Pathology, n (%)				χ²=30.39	< 0.001
Uncomplicated	181 (54.68)	108 (71.05)	73 (40.78)		
Complicated	150 (45.32)	44 (28.95)	106 (59.22)		
Surgical method, n (%)				χ²=3.15	0.208
Laparoscopy	209 (63.53)	103 (68.67)	106 (59.22)		
Laparotomy	79 (24.01)	31 (20.67)	48 (26.82)		
Conversion	41 (12.46)	16 (10.67)	25 (13.97)		

t: t-test, χ²: Chi-square test

*SD* Standard deviation, *BMI* Body mass index, *RLQ* Right lower quadrant, *WBC* White blood cell, *CRP* C-reactive protein, *ALP* Alkaline phosphatase, *AST* Aspartate transaminase, *ALT* Alanine aminotransferase, *TBL* Total bilirubin, *NLR* Neutrophil/lymphocyte ratio

Table 2 also provides information and comparison between patients with and without appendicoliths. Migratory right lower abdominal pain was significantly more common in patients with appendicoliths (53.37%) compared to those without (41.06%) (χ²=4.96, *p* = 0.026). Nausea and vomiting were more frequently reported by patients with appendicoliths (58.99%) than those without (40.40%) (χ²=11.30, *p* < 0.001). In lab tests, patients with appendicoliths had higher white blood cell counts (12.66 ± 4.50 vs. 10.79 ± 4.55, *p* < 0.001) and neutrophil counts (10.71 ± 4.33 vs. 8.57 ± 4.52, *p* < 0.001). C-reactive protein (CRP) levels were lower in patients with appendicoliths (21.62 ± 9.76 vs. 24.49 ± 15.96, *p* = 0.046). AST levels were higher in patients with appendicoliths (284.28 ± 96.31 vs. 263.49 ± 87.17, *p* = 0.045). Sodium levels were lower in patients with appendicoliths (138.71 ± 10.65vs. 140.67 ± 2.85, *p* = 0.028). CRP/Albumin and NLR were higher in patients with appendicoliths (*p* = 0.037, *p* = 0.011 respectively). In terms of pathology, complicated appendicitis was significantly more common in patients with appendicoliths (59.22%) compared to those without (28.95%) (χ²=30.39, *p* < 0.001).

In multivariable analysis of factors associated with presence of appendicoliths (Table 3), 4 independent factors were found to be associated with appendicoliths, including nausea or vomiting (OR = 1.71, 95% CI: 1.03 ~ 2.83, *p* = 0.037), lower sodium levels (OR = 0.91, 95% CI: 0.84 ~ 0.99, *p* = 0.041), complicated appendicitis(OR = 1.88, 95% CI: 1.04 ~ 3.40, *p* = 0.036) and higher neutrophilia (NEU%) (OR = 1.03, 95% CI: 1.01 ~ 1.05, *p* = 0.031).

**Table 3:** Univariable and multivariable analysis of factors associated with presence of appendicoliths

Variables	Univariable analysis					Multivariable analysis				
	β	S.E	Z	*P*	OR (95%CI)	β	S.E	Z	*P*	OR (95%CI)
Migration of pain	0.50	0.22	2.22	0.026	1.64 (1.06 ~ 2.55)	0.42	0.26	1.63	0.102	1.52 (0.92 ~ 2.51)
Nausea or vomiting	0.75	0.23	3.34	< 0.001	2.12 (1.36 ~ 3.30)	0.54	0.26	2.08	0.037	1.71 (1.03 ~ 2.83)
Sodium	−0.13	0.04	−3.35	< 0.001	0.88 (0.81 ~ 0.95)	−0.09	0.04	−2.04	0.041	0.91 (0.84 ~ 0.99)
AST	−0.02	0.01	−1.89	0.059	0.98 (0.96 ~ 1.00)	−0.02	0.01	−1.43	0.153	0.98 (0.96 ~ 1.01)
Pathology										
Uncomplicated					1.00 (Reference)					1.00 (Reference)
Complicated	1.27	0.23	5.41	< 0.001	3.56 (2.25 ~ 5.65)	0.63	0.30	2.09	0.036	1.88 (1.04 ~ 3.40)
CRP/Albumin	0.11	0.05	2.03	0.042	1.11 (1.01 ~ 1.23)	−0.07	0.06	−1.10	0.271	0.93 (0.82 ~ 1.06)
Neutrophilia (%)	0.05	0.01	4.57	< 0.001	1.05 (1.03 ~ 1.07)	0.03	0.01	2.16	0.031	1.03 (1.01 ~ 1.05)

β Regression coefficient, S.E. Standard error, Z Z-statistic, OR Odds Ratio, CI ConfidenceInterval, AST Aspartate transaminase, CRP C-reactive protein

Univariable and multivariable analyses (Table 4) revealed three factors associated with complicated appendicitis between patients with appendicoliths. These included appendiceal diameter (OR = 1.20; 95% CI: 1.03 ~ 1.40), moderate-severe fat stranding (OR = 17.61; 95% CI: 3.19 ~ 97.33), periappendiceal air (OR = 9.78; 95% CI: 1.17 ~ 81.46).

**Table 4:** Univariable and multivariable analysis of factors associated with complicated appendicitis between patients with appendicoliths

Variables	Univariable analysis					Multivariable analysis				
	β	S.E	Z	*P*	OR (95%CI)	β	S.E	Z	*P*	OR (95%CI)
Appendiceal diameter	0.25	0.07	3.52	< 0.001	1.28 (1.12 ~ 1.47)	0.18	0.08	2.33	0.020	1.20 (1.03 ~ 1.40)
Appendiceal length	0.20	0.12	1.73	0.083	1.22 (0.97 ~ 1.53)					
Appendicolith diameter	0.13	0.05	2.51	0.012	1.14 (1.03 ~ 1.27)	0.05	0.07	0.75	0.455	1.05 (0.92 ~ 1.20)
Appendicolith location										
Proximal					1.00 (Reference)					
Middle	0.17	0.46	0.36	0.721	1.18 (0.48 ~ 2.93)					
Distal	0.73	0.65	1.13	0.259	2.08 (0.58 ~ 7.46)					
Appendicolith number										
Single					1.00 (Reference)					
Multiple	−0.29	0.47	−0.62	0.537	0.75 (0.30 ~ 1.87)					
Sludge	−0.45	0.55	−0.83	0.406	0.63 (0.22 ~ 1.86)					
Fat stranding										
None					1.00 (Reference)					1.00 (Reference)
Mild	1.88	0.80	2.35	0.019	6.53 (1.36 ~ 31.36)	1.52	0.82	1.87	0.062	4.59 (0.93 ~ 22.70)
Moderate-severe	3.63	0.85	4.28	< 0.001	37.62 (7.13 ~ 198.42)	2.87	0.87	3.29	0.001	17.61 (3.19 ~ 97.33)
Extraluminal fluid										
None					1.00 (Reference)					
Minute	1.09	0.52	2.09	0.036	2.99 (1.07 ~ 8.33)	−0.31	0.68	−0.45	0.651	0.74 (0.20 ~ 2.78)
Little	2.69	0.76	3.53	< 0.001	14.72 (3.31 ~ 65.41)	0.82	0.88	0.93	0.353	2.26 (0.40 ~ 12.65)
Large	3.19	1.04	3.06	0.002	24.32 (3.15 ~ 187.88)	1.93	1.12	1.73	0.084	6.90 (0.77 ~ 61.82)
Periappendiceal air	2.89	1.04	2.76	0.006	17.94 (2.32 ~ 138.96)	2.28	1.08	2.11	0.035	9.78 (1.17 ~ 81.46)
Small bowel dilatation	1.70	1.09	1.57	0.117	5.49 (0.65 ~ 46.08)					

*β* Regression coefficient, *S.E.* Standard error, *Z* Z-statistic, *OR* Odds Ratio, *CI* Confidence Interval

## Discussion

Previous studies have shown that approximately 20%−40% of patients with acute appendicitis have appendicolith on CT [2, 3, 5]. In our study, 179 out of 331 patients with acute appendicitis had appendicoliths, resulting in an appendicolith-associated appendicitis proportion of 54%, higher than previous studies. The diagnosis of appendicolith in this study included imaging data, intraoperative findings, and postoperative pathology, resulting in a higher detection rate. This may be related to the fact that most cases of appendicitis were emergency admissions, with patients presenting with more severe conditions and longer symptom duration. Additionally, this study included only surgical patients, which may also reflect the subjective bias of surgeons who believe that the presence of appendicoliths warrants surgery.

In our study, the identification of appendicoliths was not solely dependent on preoperative CT but also integrated intraoperative observations and postoperative pathological examinations. CT is the preferred preoperative imaging modality for appendicolith detection, but it has an inherent false-negative rate (14.5% in our study, with 26 of 179 appendicolith-positive patients missed by preoperative CT); these overlooked appendicoliths are mostly small (2–4 mm in diameter, accounting for 76.9% of false-negative cases). Excluding these patients would incorrectly classify them into the “appendicolith-negative group,” leading to baseline bias between groups. By including these cases, we ensure that the “appendicolith-negative group” truly represents patients without appendicoliths, and the CT-based clinical decision rules established accordingly are more trustworthy in real-world practice, as they fully account for CT limitations and avoid misguidance caused by unrecognized appendicoliths.

Most studies have found that appendicitis with appendicolith presents more severe clinical symptoms, higher rates of appendiceal perforation, and more severe pathological findings, with a higher tendency towards complicated appendicitis [13, 18, 23, 24]. Our study found that the rate of complicated appendicitis in patients with appendicolith is approximately twice that of those without appendicolith, with more severe pathological findings (*P* < 0.001), which is similar to Kaewlai et al. findings [22].

Multivariable analysis identified several factors significantly associated with the presence of appendicolith. Nausea and vomiting, lower sodium levels, complicated appendicitis, and higher neutrophil percentages were all significant independent predictors. These symptoms, coupled with elevated neutrophil counts and the presence of complicated appendicitis, suggest a more inflammatory and possibly severe presentation in patients with appendicoliths. Lower sodium levels were also associated with appendicolith, potentially related to electrolyte disturbances caused by vomiting, though the clinical relevance requires further investigation. The higher incidence of these clinical signs underscores the need for heightened clinical suspicion and potentially more aggressive management in these patients.

For many individuals, appendicoliths are incidental findings, identified by computed tomography (CT) and autopsy series in a variable proportion (3%−25%) of those without appendicitis [5, 19, 20]. In patients with uncomplicated appendicitis, previous reports show the prevalence of appendicolith in histologically confirmed uncomplicated appendicitis ranging from 13.8% to 44.0% [17, 22, 23]. Our study found a prevalence of appendicolith in 40.3% of patients with uncomplicated appendicitis (73 out of 181 patients), similar to previous studies. The detection rate of appendicoliths in uncomplicated appendicitis is not low, highlighting the importance of recognizing the clinical severity of appendicitis with appendicolith.

We compared the characteristics of the appendix, appendicoliths, and certain CT features in complicated and uncomplicated appendicitis within the appendicolith group. Among patients with appendicoliths, several factors were associated with the development of complicated appendicitis. Increased appendiceal diameter, moderate to severe fat stranding, and periappendiceal air were all significant predictors. The presence of these risk factors should prompt clinicians to consider early surgical intervention to prevent complications.

Appendiceal diameter is an important method for assessing appendicitis, with a diameter >7 mm commonly indicating appendicitis. Appendicitis causes luminal enlargement, with significant congestion and edema of the appendiceal wall, thus correlating the severity of appendicitis with appendiceal diameter. In a retrospective study [15] of 81 patients with acute uncomplicated appendicitis treated non-surgically, a logistic regression model adjusted for demographics, comorbidities, admission vital signs, and admission laboratory values showed that patients with an appendiceal diameter of 13 mm or larger were more likely to fail antibiotic treatment alone (OR = 17.55 [95% CI, 1.30–237.28.30.28]). In our study, increased appendiceal diameter was an independent risk factor for complicated appendicitis in patients with appendicolith (OR 1.20 [95% CI, 1.03–1.40], *P* = 0.02), with a cut-off of 13.1 mm, consistent with the previous study.

However, appendiceal diameter alone cannot determine the severity of appendicitis. When the appendix perforates, intraluminal pressure drops sharply, causing secretion overflow, which may reduce appendiceal diameter. Periappendiceal air is a common CT sign of appendiceal perforation and an important independent risk factor. In our study, moderate-to-severe fat stranding was an independent risk factor for identifying complicated appendicitis, consistent with findings from other studies [21].

Previous studies have shown that the size and location of appendicoliths are associated with complications [13, 25–27]. The size, location, and number of appendicoliths were also examined, though they were not found to be associated with a higher risk of complicated appendicitis. The location of appendicoliths (proximal vs. distal) did not show a significant association with complicated appendicitis in the multivariate analysis, indicating that other factors play a more critical role in the progression to complicated appendicitis. Although the diameter of appendicoliths did not show a significant association in multivariate analysis, the trend suggests that size could still play a role in clinical outcomes and warrants further investigation.

Therefore, we found that the basic features of appendicoliths are not generally associated with complicated appendicitis, suggesting that other CT findings are needed to determine the clinical severity of appendicitis with appendicoliths. Additionally, clinical findings and laboratory tests should be considered to rule out uncomplicated appendicitis, which we will study in the next step.

The findings of this study have several important clinical implications. First, comparing key clinical and laboratory predictors of appendicitis with and without appendicoliths can enhance diagnostic accuracy and inform clinical decision-making. Second, the factors associated with complicated appendicitis among patients with appendicoliths can guide management strategies, potentially leading to earlier and more aggressive interventions to prevent complications.

This study has several limitations. Firstly, the retrospective nature of the study design may introduce selection bias and limit the ability to establish causality. Secondly, the study was conducted at a single center. Single-center cohort limits generalizability, as CT scanning protocols and surgical decision-making may vary across institutions. Thirdly, many patients with appendicitis were excluded due to the use of alternative diagnostic methods, such as preoperative ultrasound or outside-hospital CT, or because they directly underwent surgery. Fourthly, CT feature assessment (e.g., fat stranding severity) was subjective, despite consensus resolution of discrepancies. Lastly, variables such as appendiceal wall thickness and mucosal enhancement defect (reported as predictors in other studies [21]) were not included in our analysis, which may have missed additional predictive factors. These factors may affect the proportion of patients with and without complications and limit the generalizability of the findings to other populations or settings. Future studies should aim to validate these findings in larger, multicenter cohorts and explore the underlying mechanisms linking appendicoliths to complicated appendicitis, including the size, location, and number of appendicoliths.

## Conclusion

This study provides valuable insights into the clinical characteristics and factors associated with appendicoliths and complicated appendicitis. The identified predictors can enhance clinical assessment and guide management strategies, potentially improving outcomes for patients with appendicitis. Further research is needed to validate these findings and explore the mechanisms underlying the observed associations.

## Data Availability

The data that support the findings of this study are available on request from the corresponding author, upon reasonable request.

## Abbreviations

CT: Computed tomography
OR: Odd ratio
NOM: Nonoperative management
SD: Standard deviation
BMI: Body mass index
RLQ: Right lower quadrant
WBC: White blood cell
CRP: C-reactive protein
ALP: Alkaline phosphatase
AST: Aspartate transaminase
ALT: Alanine aminotransferase
TBL: Total bilirubin
NLR: Neutrophillymphocyte ratio
β: Regression coefficient
S.E.: Standard error
Z: Z-statistic
